# Krebs Von den Lungen-6 as a predictive indicator for the risk of secondary pulmonary fibrosis and its reversibility in COVID-19 patients

**DOI:** 10.7150/ijbs.58825

**Published:** 2021-04-10

**Authors:** Mingshan Xue, Teng Zhang, Hao Chen, Yifeng Zeng, Runpei Lin, Yingjie Zhen, Ning Li, Zhifeng Huang, Haisheng Hu, Luqian Zhou, Hui Wang, Xiaohua Douglas Zhang, Baoqing Sun

**Affiliations:** 1 State Key Laboratory of Respiratory Disease, National Clinical Research Center for Respiratory Disease, Guangzhou Institute of Respiratory Health, First Affiliated Hospital of Guangzhou Medical University, Guangzhou 510120, China.; 2 Faculty of Health Sciences, University of Macau. Taipa, Macau, China.; 3 Department of Allergy, Tongji Hospital, Tongji Medical College, Huazhong University of Science and Technology, Wuhan 430030, China.; 4 Department of Medical Laboratory, The Central Hospital of Wuhan, Tongji Medical College, Huazhong University of Science and Technology, Wuhan 430014, China.

**Keywords:** Krebs von den Lungen-6, Coronavirus disease 2019, Pulmonary fibrosis

## Abstract

Dysregulated immune response and abnormal repairment could cause secondary pulmonary fibrosis of varying severity in COVID-19, especially for the elders. The Krebs Von den Lungen-6 (KL-6) as a sensitive marker reflects the degree of fibrosis and this study will focus on analyzing the evaluative efficacy and predictive role of KL-6 in COVID-19 secondary pulmonary fibrosis. The study lasted more than three months and included total 289 COVID-19 patients who were divided into moderate (n=226) and severe groups (n=63) according to the severity of illness. Clinical information such as inflammation indicators, radiological results and lung function tests were collected. The time points of nucleic acid test were also recorded. Furthermore, based on Chest radiology detection, it was identified that 80 (27.7%) patients developed reversible pulmonary fibrosis and 34 (11.8%) patients developed irreversible pulmonary fibrosis. Receiver operating characteristic (ROC) curve analysis shows that KL-6 could diagnose the severity of COVID-19 (AUC=0.862) and predict the occurrence of pulmonary fibrosis (AUC = 0.741) and irreversible pulmonary fibrosis (AUC=0.872). Importantly, the cross-correlation analysis demonstrates that KL-6 rises earlier than the development of lung radiology fibrosis, thus also illuminating the predictive function of KL-6. We set specific values (505U/mL and 674U/mL) for KL-6 in order to assess the risk of pulmonary fibrosis after SARS-CoV-2 infection. The survival curves for days in hospital show that the higher the KL-6 levels, the longer the hospital stay (P<0.0001). In conclusion, KL-6 could be used as an important predictor to evaluate the secondary pulmonary fibrosis degree for COVID-19.

## Introduction

The outbreak of COVID-19 has become the research hotspot nowadays and its pathological mechanism of intra- or extra-pulmonary manifestations has been gradually clarified 1, 2. The alveolar epithelial injury is the main characteristic for COVID-19 which is caused by direct viral damage, inflammatory cytokine storm, drug effects or barotropic oxygen therapy and this condition is overlapping rather than separated 3, 4. There are about 1-2 million people who are already recovered but remained with pulmonary fibrosis in varying degree 3. In the study of secondary disease for COVID-19, the visible fibrosis lung remodeling that generates restrictive lung function abnormalities after inflammatory storm has attracted attention from clinicians 5-8. It is not unusual for the manifestation of pulmonary fibrosis appearing in acute infectious disease 3. Some COVID-19 patients recover from acute respiratory distress syndrome but still have hypoxemia and pulmonary fibrosis which are found by radiology 9. The long-term irreversible sequela of pulmonary fibrosis could significantly damage ventilation function in the elderly and lead to poor prognosis for patients 3, 8. Therefore, the reversibility of secondary pulmonary fibrosis in COVID-19 is worthy of our attention.

KL-6, which overexpresses in the damaged or repaired type 2 alveolar epithelium and mainly reflects the degree of interstitial lung impairment, might be helpful for the evaluation of pulmonary fibrosis for COVID-19 10. Current studies also find the increase of KL-6 in COVID-19 patients and indicate that the KL-6 is correlated with the severity of COVID-19, which can be used as a diagnostic assessment index 11, 12. According to radiological reports, the severe patients have larger irregular pulmonary fibrosis lesions which are difficult to absorb completely 13, 14. Therefore, the degree of activation of lung fibroblasts may be different for patients with varying severity 15. Many studies have indicated that KL-6 could be a measure for the risk evaluation of COVID-19^11^, ^16^-^18^. However, there is no consensus standard at present for the risk evaluation of lung pulmonary fibrosis on COVID-19. Thus, we attempt to establish a tentative criteria of KL-6-Fibrosis-Reversibility risk assessment for COVID-19. This study adopts KL-6 to assess the risk of secondary pulmonary fibrosis in patients with COVID-19 and explore whether KL-6 can estimate the reversibility of fibrosis to evaluate the prognosis.

## Materials and methods

### Participants

A total of 289 COVID-19 patients were involved in this study from the First Affiliated Hospital of Guangzhou Medical University (n = 21, moderate/severe=7/14) and Wuhan Central Hospital (n = 268, moderate/severe=219/49). The COVID-19 patients were confirmed by reverse transcriptase-polymerase chain reaction (RT-PCR) test and divided into two groups as moderate (n=226) and severe (n=63) groups according to the latest diagnosis criteria 19. The negative standard of RT-PCR in COVID-19 patients was defined as the results showing negative after more than three tests at different times.

This research complied with the ethical standards determined by the ethics committee of First Affiliated Hospital of Guangzhou Medical University, with the ethical approval number 2020-77 and 2020-85.

### Definition of moderate and severe COVID-19 19-22

Criteria of moderate patients: The COVID-19 patients with respiratory symptoms such as cough, sputum, polypnea, dyspnea or fever, besides of radiology reports showing the manifestation of pneumonia. Criteria of severe patients: 1. Respiratory rate>30 times/min; 2. In resting state, oxygen saturation < 93%; Chest radiology progression > 50% within 24-48 h. The patients require intensive care that includes non-invasive or invasive mechanical ventilation.

### Chest radiology detection

The study adopted high-resolution CT (1mm slice) to observe the pulmonary lesions. Digital automatic quantitative assessment system was adopted in this study, and the ratio of lesion area which included ground glass shadow, patch shadow, consolidation, alveolar exudation, and interlobular involvement to total lung area was used to evaluate the pulmonary abnormity. All the statistical data of radiology results in this study were obtained from high-resolution CT. 3 radiologists with more than 5 years of working experience participated in this research to perform qualitative identification for the lesions. Meanwhile, the fibrosis characteristic of irregular linear and reticular opacities was also observed and evaluated. The specific method formulation was referred from Wong et al 2020 23, Li et al 2020 24, and Xie et al 2020 25. Because of the limitations in the realistic conditions, the high-resolution CT data was not completely consistent in the time and frequency of testing for each patient. After calculating all test data, we matched all the time point and then observed the overall linear trend.

### Definition of secondary pulmonary fibrosis for COVID-19

Secondary pulmonary fibrosis is the condition in which patients without previous interstitial lung diseases are shown with partial or extensive new fibrous lesion in radiography during the progression of COVID-19 3, 26, 27. The reversibility of fibrosis is determined by the comparison between fibrosis shadows from the first and the last radiographic examinations. Irreversible fibrosis is defined as no significant changes for the focal fibrosis. Reversible fibrosis is defined as significant reticular shadow absorption without septal thickening.

### KL-6 detection

Patients' serum was collected (3000rpm, 5min, room temperature) and stored at -20℃. The KL-6 level was detected by automatic biochemical analyzer for the change of absorbance caused by the emulsion agglutination reaction. The measurement range is 50-6000 U/mL.

### Data extraction

All clinical data were extracted from the patient's electronic medical records. Demographic data and the KL-6, blood cell, inflammatory factor, blood gas, and SARS-CoV-2 nucleic acid test results were extracted. In this study, a KL-6 kit (Shanghai Medconn Diagnostics Technology Co., Ltd. China) was used to detect changes in the sample absorbance and analyze the concentration level of samples using a fully automatic biochemical analyzer (HITACHI-7180, Ltd. Japan).

### Statistical analyses

Continuous variables in this study were presented as median (interquartile range [IQR]) and categorical variables were presented as number (frequency). The groups were compared using the Mann-Whitney U test for continuous variables and the Chi-square test (or Fisher's exact test if required) for categorical variables. A p value of 0.05 or less was considered statistically significant. Spearman's correlation analysis was used to estimate the correlation between the groups. Cross-correlation function (CCF) testing was applied to explore the correlation between the trend of KL-6 and radiology result. Kaplan-Meier curves were used to estimate the cumulative incidence of discharge events in patients with different KL-6 levels. The difference of these Kaplan-Meier curves was analyzed using the log-rank test. The phase relationship was examined by determining the time lag at which the value of the CCF was maximal. The receiver operating characteristic (ROC) curve analysis was used to evaluate the diagnostic efficacy of KL-6. Statistical analyses were performed using R software, version 4.0.0 (R Core Team) and GraphPad Prism version 8.0.2 (GraphPad Software, San Diego, CA, USA).

## Results

### Patients characteristics

As shown in Table 1, 226 patients with moderate COVID-19 and 63 patients with severe COVID-19 are included. The KL-6, CRP, Leukocyte count and Neutrophil count are significantly higher in patients with severe COVID-19 compared with patients with moderate COVID-19, while the lymphocyte count is lower. Analysis of the correlation between KL-6 levels and blood gas indicators in COVID-19 patients finds that KL-6 is significantly correlated with respiratory index (RI) and artery-alveolar partial pressure difference of oxygen (PA-aO2) (Figure S1). The distribution of KL-6 levels in moderate and severe patients and the ROC curve of KL-6 to diagnose the severity of COVID-19 are shown in Figure S2. The AUC is 0.862(0.809-0.914).

### Chest radiology reports

The degree of lung involvement and damage in COVID-19 patients with different severity is different (Table 2). The ground glass opacities and the shown patch are the most common in the acute phase of COVID-19 onset which is the characteristic manifestation of exudative change of lung infectious disease and some patients have pleural effusion. The ratio of linear opacity and thickened interlobular septum is high in severe patients, which is a characteristic of fibrosis.

### The occurrence of secondary pulmonary fibrosis in COVID-19 patient

Among 289 COVID-19 patients (moderate/severe = 226/63) included in this study, 114 patients develop secondary pulmonary fibrosis in varying degrees, including 58 cases of moderate (50.9%) and 56 cases of severe (49.1%), and 175 patients do not develop secondary pulmonary fibrosis, including 168 cases of moderate (96.0%) and 7 cases of severe (4.0%). In addition, the reversibility of pulmonary fibrosis (based on residual fibrous lesions revealed in the last radiology when the patient was discharged) is also evaluated. Among the 114 patients with pulmonary fibrosis, 80 patients are reversible fibrosis, of which 32.5% are severe patients (n=26), and 34 patients are irreversible fibrosis, of which 88.2% are severe Patients (n=30) (Table S1).

The clinical information for COVID-19 patients classified by whether pulmonary fibrosis has occurred is showed in Table 3. The results show that the KL-6 and CRP are significantly higher in COVID-19 patients with pulmonary fibrosis compared with patients without pulmonary fibrosis. Although the white blood cell and neutrophil counts are higher in COVID-19 patients with pulmonary fibrosis, the lymphocyte count is lower.

**Figure 1:**
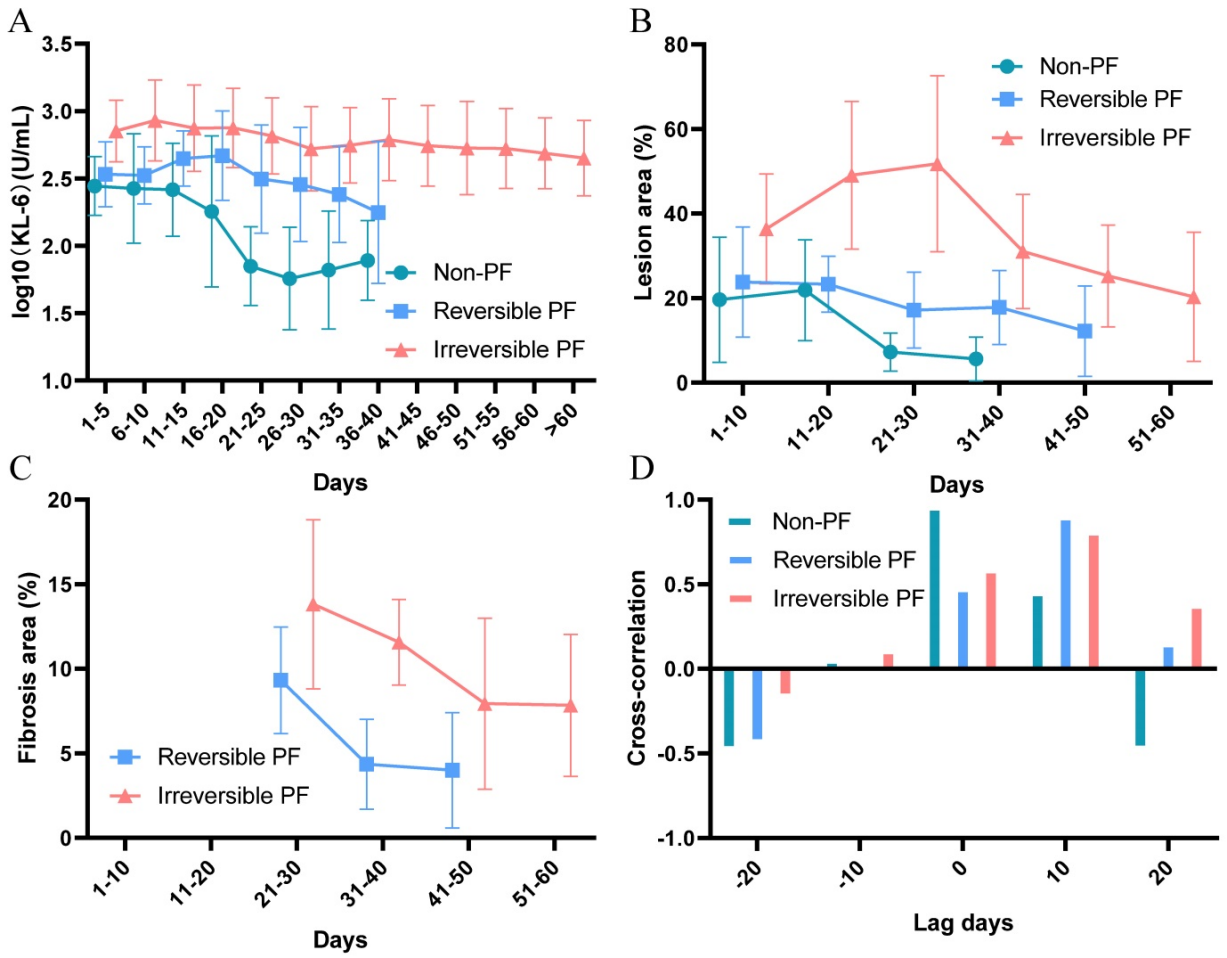
** The trend of KL-6 and high-resolution CT in COVID-19 patients.** (A). The trend of KL-6 in COVID-19 patients without fibrosis, with reversible fibrosis and with irreversible fibrosis. (B). The trend of lesion area (%) shown by CT results in COVID-19 patients without fibrosis, with reversible fibrosis and with irreversible fibrosis. (C). The trend of fibrosis area (%) in COVID-19 patients with reversible fibrosis and with irreversible fibrosis. The characteristic of irregular fibrotic shadow (interlobular septa thickened, reticular or linear opacities pattern) appears clearly about 20 days after symptom onset of COVID-19. Spagnolo et al 2020 3 also indicates this manifestation appearing after the onset of symptoms. At the same time, in the acute phase, extensive ground glass and patchy opacity may also obscure the fibrosis manifestation. Therefore, we use the time point of 20 days as the starting point for the evaluation of pulmonary fibrosis lesions. (D). The cross-correlation function between the trend of KL-6 and the trend of CT.

Figure S3A show that the KL-6 level decreases gradually, and the lesion also diminishes in area and is absorbed during the in-hospital treatment in moderate COVID-19 patients while the KL-6 in severe patients maintains at high level. The high-resolution CT results (Figure S3B and Figure 1B) show the total lesion area, which includes not only the fibrotic manifestations, but also new lesions automatically counted by the quantification system. The high-resolution CT does not drop to 0 because there are some patients who meet the conditions for discharge still left with irreversible secondary pulmonary fibrosis. In addition, some patients do not recheck the chest high-resolution CT before discharge, hence the exudative lesions shown in the last result are not fully absorbed.

In Figure 1A, the KL-6 is at highest level in irreversible pulmonary fibrosis patients. The Figure 1B also shows that patients in the irreversible group also have a higher level of lung exudation at the beginning of the disease progression. We separately review the progressive trend of pulmonary fibrosis in patients of reversible and irreversible (Figure 1C) which indicates that the irreversible group has higher degree of pulmonary fibrosis. We further calculate the cross-correlation between the trend of high-resolution CT and KL-6 levels (Figure 1D). The correlation is highest when the lag days of KL-6 is 0, 10 and 10 in patients without pulmonary fibrosis, with reversible fibrosis and with irreversible fibrosis. (P<0.05, r=0.962, 0.903 and 0.813, respectively), which means the KL-6 level increases earlier than the fibrosis lesion emerges.

### Diagnostic efficiency analysis of KL-6 for fibrosis and its reversibility in COVID-19 patients

We collect the first KL-6 test value of the patient after admission. The KL-6 levels in patients with pulmonary fibrosis are significantly higher than those without pulmonary fibrosis and the KL-6 levels in patients with irreversible fibrosis are significantly higher than those with reversible fibrosis (P<0.01). The ROC curve shows that the first KL-6 test value after admission can predict the occurrence of fibrosis and irreversible fibrosis with the AUC of 0.741 (0.677,0.804) and 0.872(0.794,0.951), respectively. The optimal threshold distinguishes COVID-19 patients without fibrosis and with fibrosis was 505 U/mL, with a sensitivity of 0.535 and specificity of 0.914, and the optimal threshold to distinguish COVID-19 patients with reversible fibrosis and with irreversible fibrosis is 674 U/mL, with a sensitivity of 0.824 and specificity of 0.838.

We also collected the KL-6 values from 56 patients after their PCR test results turned negative. The KL-6 levels after the PCR-negative in patients with irreversible fibrosis is significantly higher than those with reversible fibrosis and those without fibrosis (P<0.05) (Figure S4). In addition, there are 30 patients with pulmonary fibrosis, of which 18 patients (60.0%) have KL-6 levels higher than the threshold of 505 U/mL. There are 20 patients with irreversible pulmonary fibrosis, of which 13 patients (65.0%) have KL-6 levels higher than the threshold of 674 U/mL (Table S2).

### The relationship between KL-6 and length of hospital stay

**Figure 3:**
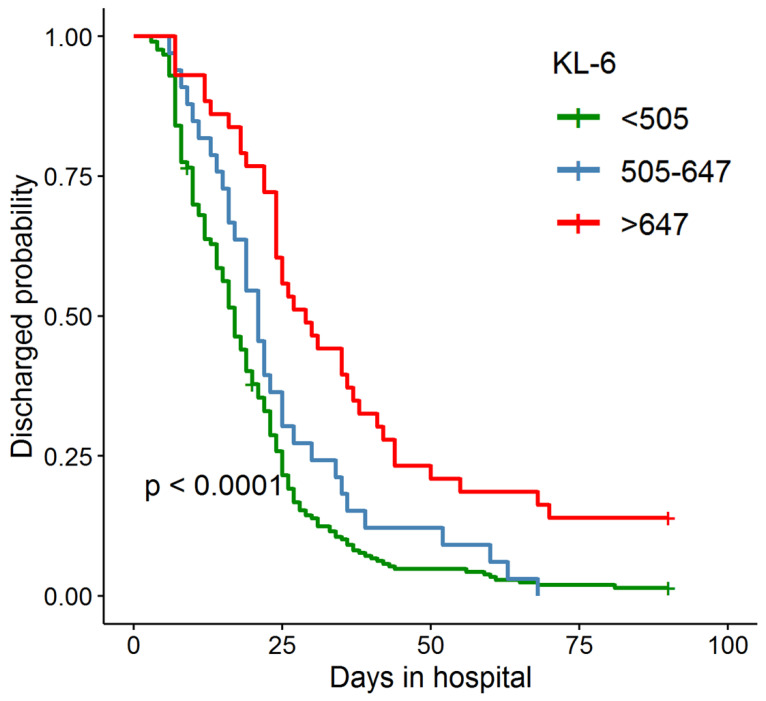
**Survival curve of days in hospital for COVID-19 patients.** The patients are grouped according to the first KL-6 test after admission. The green line: KL-6<505U/mL; The blue line: 505U/mL<KL-6<674U/mL; The red line: KL-6>674U/mL.

After 90 days of hospitalization, 278 patients recovered and were discharged, 2 patients died, and 9 patients were still hospitalized. Base on the days in hospital, we draw the survival curves of COVID-19 patients (Figure 3). The patients are divided into three groups according the first KL-6 test values. Log-rank test shows that the difference in days in hospital between the three groups is significant. The higher the KL-6, the longer the days in hospital.

## Discussion

In this COVID-19 research, KL-6 shows a higher expression level especially in the severe patient group and parallels with the disease progression and remission. The significant correlation between KL-6 and the inflammatory indicators, along with the linear analysis result showing similar trend for KL-6 and lesion area ratio, indicates that KL-6 is closely involved in the pathological mechanism of COVID-19. In addition, KL-6 can be used as an indicator to assess the risk of pulmonary fibrosis in patients with COVID-19 and to assess the reversibility of fibrosis.

The increased KL-6 is found in the COVID-19 patients and thus we speculate it could be employed to assess the acute infectious diseases with secondary pulmonary fibrosis. The correlation analysis with the blood gas and inflammatory index of CRP, PCT and interleukins could prove KL-6 is associated with the pulmonary ventilation and the inflammation level. Meanwhile, in our research, pulmonary radiology shows varying degrees of unilateral or bilateral lung exudation mostly present as ground-glass or patchy shadow at the early stage of COVID-19 patients. Especially in severe COVID-19, the lung exudation has larger area and fast progression, and the whole lung is involved in most cases. The primary manifestation of COVID-19 is diffuse alveolar injury, accompanied by numerous lung epithelial cells which are proliferating, atrophic, exfoliating, or squamous metaplasia in acute phase, which result in the destruction of the alveolar epithelial barrier, massive lung exudation and the large release of the KL-6 28, 29. Especially for the severe COVID-19 patients, this pathological process can be more severe and lead to more KL-6 being released into the blood through the damaged alveolar basement membrane 30, 31. Excessive deposition of extracellular matrix during lung repair and remodeling would eventually generate residual pulmonary fibrotic lesions 32. Besides, we find that KL-6 is increased earlier than the time of imaging lesions occur which could prove that the visible organic damage is later than KL-6. This suggests that KL-6 may act as a predictor of COVID-19 lung injury.

However, it is important to note that the significant correlation between KL-6 and pulmonary fibrosis is not found in all COVID-19 patients. In the early stage of disease, the activation of lung epithelium repair mechanism is almost simultaneous when large-area pulmonary injury occurs 33. We consider that this tendency of pulmonary fibrosis also causes the increase of KL-6. The potential nonorganic lesion assessment is always difficult to achieve at present by radiological visualization. Similarly, in some COVID-19 patients with secondary fibrosis, KL-6 level is not significantly increased, possibly because the time we found pulmonary fibrous change is later than the time when KL-6 releases in large amount and activates the fibrosis repair mechanism. Nevertheless, radiological assessment remains a necessary and visualized quantitative method for assessing the level of lung injury in COVID-19.

Among the COVID-19 patients in this study, 39.4% (n=114) has varying degrees of fibrotic changes. The level of KL-6 in patients with irreversible fibrosis after negative PCR is significantly higher than that in patients with reversible fibrosis and without fibrosis. Undoubtedly, most severe patients have a higher KL-6 level and a higher risk of secondary pulmonary fibrosis compared with moderate patients. If the KL-6 level remains above 505 U/mL for a long time after the negative phase, the patients is prone to the progression of secondary fibrosis. A decrease of KL-6 in negative stage does not mean that fibrosis is reversible, because the KL-6 level increases to over 673U/mL during positive period, which indicates that the patient has developed irreversible fibrosis already. In addition, it is also necessary to focus on whether the pulmonary fibrosis is in a progressive state or the old lesion manifestation, because the old lesion manifestation was irreversible and KL-6 level in patients with old lesions may not increase. Therefore, we should first pay attention to whether the KL-6 level of patients with secondary pulmonary fibrosis exceeds 674 U/mL, which reflects irreversible fibrosis during the process of SARS-CoV-2 infection, and then judge whether the KL-6 level decreases after PCR turns negative, which reflects gradual absorption of pulmonary fibrosis.

Further, we classify the COVID-19 patients based on their KL-6 levels (with the thresholds of 505 and 674 U/mL) and find that patients with higher KL-6 levels had longer hospital stays. Therefore, in addition to determining the reversibility of the disease, KL-6 levels of COVID-19 patients could also preliminarily determine the expected length of the hospitalization. It also further supports the close association between KL-6 and the progression of COVID-19.

## Conclusion

The KL-6 that is associated with the degree of alveolar epithelial injury could be used as a novel diagnosis and prediction indicator for COVID-19. Furthermore, two specific values of KL-6 are calculated to predict the risk of the secondary pulmonary fibrosis and its irreversibility. These values can be used as an accurate indicator to reflect the prognosis. In addition, we believe that the evaluation efficiency of KL-6 should be combined with the course events, and multi-point KL-6 data analysis may more accurately evaluate the trend of pulmonary fibrosis changes in COVID-19.

## Supplementary Material

Supplementary figures and tables.

## Figures and Tables

**Figure 2:**
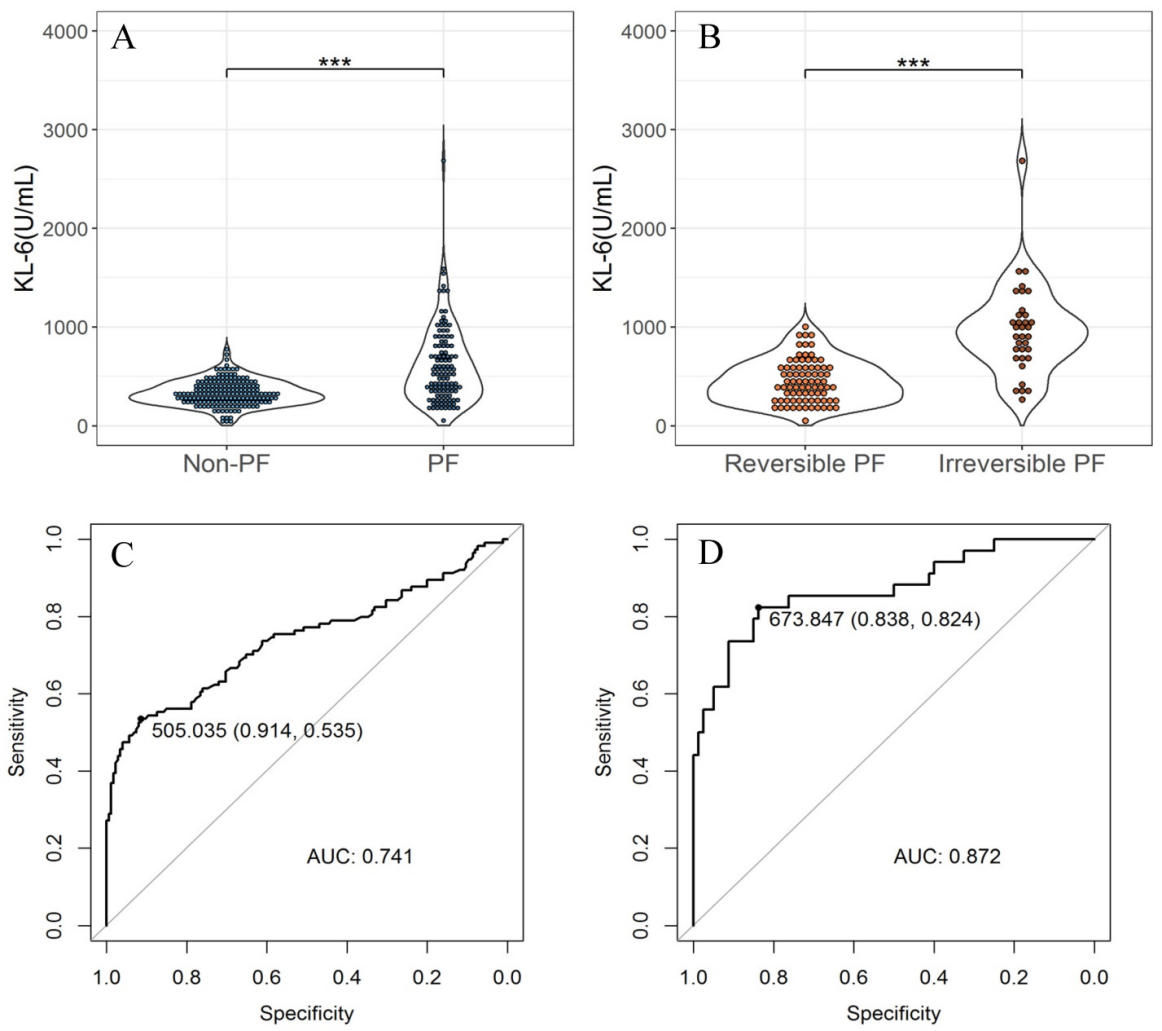
** The difference of KL-6 levels at admission in COVID-19 patients and the diagnostic efficiency of KL-6.** (A). The difference of KL-6 levels in COVID-19 patients with and without fibrosis. (B). The difference of KL-6 levels in COVID-19 patients with reversible fibrosis and with irreversible fibrosis. (C). The ROC curve illustrating performance of KL-6 to distinguish COVID-19 patients with and without fibrosis. The AUC is 0.741(0.677,0.804) and the optimal threshold is 505, with a sensitivity of 0.535 and a specificity of 0.914. (D). The ROC curve illustrating performance of KL-6 to distinguish COVID-19 patients with reversible fibrosis and with irreversible fibrosis. The AUC is 0.872(0.794,0.951) and the optimal threshold is 674, with a sensitivity of 0.824 and a specificity of 0.838. ("***" < 0.001, "**" < 0.01, "*" < 0.05).

**Table 1:** Clinical characteristics of the participants

	Moderate	Severe	p
N	226	63	
Male (%)	99 (43.8)	31 (49.2)	0.536
Age, years	56.00 [41.00, 66.00]	61.00 [54.50, 69.00]	0.007
Severe (%)			<0.001
Non-PF	168 (74.3)	7 (11.1)	
Reversible PF	54 (23.9)	26 (41.3)	
Irreversible PF	4 (1.8)	30 (47.6)	
KL-6, U/mL	322.85 [234.27, 426.90]	688.75 [469.94, 991.41]	<0.001
CRP, mg/mL	0.14 [0.06, 0.84]	1.16 [0.16, 4.65]	<0.001
***Symptom, N***			
Fever (%)	142 (62.8)	45 (71.4)	0.265
Cough (%)	122 (54.0)	46 (73.0)	0.010
Panting (%)	54 (23.9)	36 (57.1)	<0.001
Fatigue (%)	93 (41.2)	36 (57.1)	0.034
***Blood cell analysis***			
Leukocyte, 10^9^/L	4.95 [2.00, 7.07]	5.95 [4.32, 10.50]	0.013
Lymphocyte, 10^9^/L	1.55 [1.22, 1.92]	1.08 [0.80, 1.54]	<0.001
Neutrophil, 10^9^/L	3.22 [2.57, 4.31]	3.92 [2.73, 6.38]	0.004
***Biochemical detection***			
K, mmol/L	4.25 [3.94, 4.48]	4.08 [3.73, 4.36]	0.015
Na, mmol/L	140.20 [138.40, 142.15]	139.65 [136.70, 141.83]	0.154
Cl, mmol/L	104.45 [102.60, 106.30]	103.15 [99.20, 106.45]	0.056
Ca, mmol/L	2.29 [2.20, 2.38]	2.22 [2.09, 2.33]	0.031

PF: Pulmonary fibrosis; CRP: C-reactive protein.

**Table 2:** Radiologic characteristic

N (%)	Moderate	Severe
Ground glass opacities	194(85.8)	63(100.0)
Patch shadow	200(88.5)	61(96.8)
Reticular pattern	47(20.8)	24(38.1)
Linear opacities	4(1.8)	27(42.9)
Interlobular septal thickening	16(7.1)	38(60.3)
Consolidation	46(20.4)	30(47.6)
Nodule	11(4.9)	8(12.7)
Air bronchogram	1(0.4)	6(9.5)
Pleural thickening	94(41.6)	41(65.1)
Pleural effusion	53(23.5)	20(31.7)

**Table 3:** Clinical characteristics of the participants classified by the pulmonary fibrosis

	Non-PF	PF		p
		Reversible	Irreversible	
N	175	80	34	
Male (%)	76 (43.4)	33 (41.2)	21 (61.8)	0.106
Age, years	55.00 [38.00, 67.00]	59.00 [50.00, 66.00]	61.50 [55.25, 71.75]	0.025
Severe (%)	7 (4.0)	26 (32.5)	30 (88.2)	<0.001
KL-6, U/mL	318.67 [237.61, 413.11]	417.29 [265.19, 586.93]	942.51 [703.62, 1132.31]	<0.001
CRP, mg/mL	0.12 [0.05, 0.97]	0.26 [0.12, 2.39]	1.97 [0.15, 3.26]	0.003
***Symptom, N***				
Fever (%)	112 (64.0)	53 (66.2)	22 (64.7)	0.941
Cough (%)	95 (54.3)	47 (58.8)	26 (76.5)	0.056
Panting (%)	37 (21.1)	30 (37.5)	23 (67.6)	<0.001
Fatigue (%)	74 (42.3)	36 (45.0)	19 (55.9)	0.344
***Blood cell analysis***				
Leukocyte, 10^9^/L	4.66 [1.99, 6.63]	5.44 [4.57, 8.52]	5.00 [3.00, 7.51]	0.008
Lymphocyte, 10^9^/L	1.58 [1.22, 2.00]	1.46 [1.06, 1.71]	1.06 [0.87, 1.40]	<0.001
Neutrophil, 10^9^/L	3.19 [2.45, 4.20]	3.56 [2.76, 5.24]	3.79 [3.22, 5.12]	0.010
***Biochemical detection***				
K, mmol/L	4.22 [3.94, 4.44]	4.19 [3.88, 4.49]	4.14 [3.70, 4.49]	0.660
Na, mmol/L	140.10 [138.33, 141.98]	140.70 [137.70, 142.70]	139.60 [138.40, 141.50]	0.589
Cl, mmol/L	104.40 [102.70, 106.40]	104.45 [102.07, 106.25]	101.90 [98.70, 104.60]	0.013
Ca, mmol/L	2.28 [2.20, 2.38]	2.23 [2.09, 2.38]	2.30 [2.20, 2.35]	0.260

PF: Pulmonary fibrosis; CRP: C-reactive protein.

## Abbreviations

COVID-19: coronavirus disease 2019
KL-6: Krebs Von den Lungen-6
PF: pulmonary fibrosis
RI: respiratory index
PA-aO2: artery-alveolar partial pressure difference of oxygen
CRP: C-reactive protein
RT-PCR: reverse transcriptase-polymerase chain reaction
CT: computed tomography
CCF: cross-correlation function
ROC: receiver operating characteristic
AUC: area under the ROC curve
