# Supplementary material for: Krebs Von den Lungen-6 as a predictive indicator for the risk of secondary pulmonary fibrosis and its reversibility in COVID-19 patients
Source: Int J Biol Sci. 2021 Apr 10;17(6):1565–73. doi: 10.7150/ijbs.58825 (PMC8071769; doi:10.7150/ijbs.58825)
Supplement: Supplementary file 1 — Supplementary figures and tables. [file ijbsv17p1565s1.pdf]

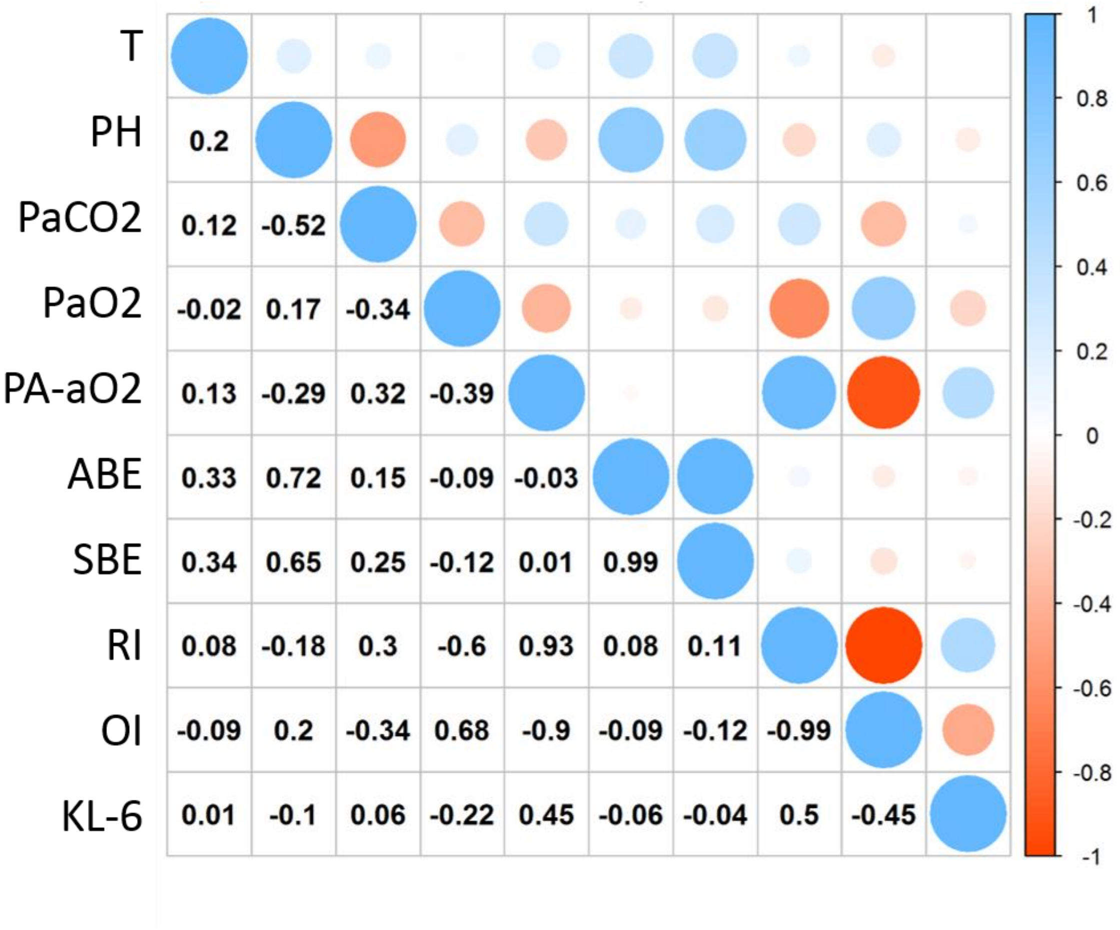

Figure S1. Correlation analysis between KL-6 and blood gas indicators in severe COVID-19 patients. (RI: Respiratory index; OI: Oxygenation index; SBE: Standard base residue; ABE: Actual base residue; PA-aO2: Artery-alveolar partial pressure difference of oxygen)

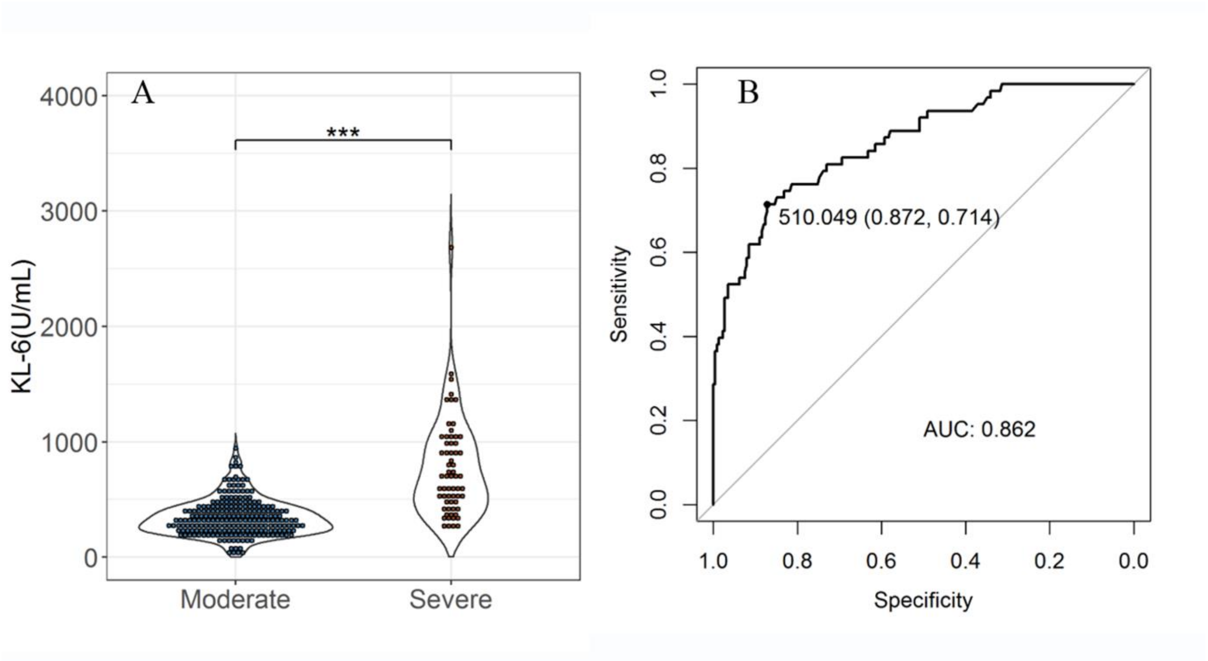

Figure S2. The difference of KL-6 between moderate patients and severe patients and the ROC curve of KL-6 to diagnose the severity of COVID-19.

Table S1. The classification of participants

	Non-PF	PF	
N(%)	175	114	
Moderate(%)	168(96.00)	58(50.88)	
Severe(%)	7 (4.00)	56(49.12)	
		Reversible	Irreversible
N(%)		80	34
Moderate(%)		54(67.50)	4(11.76)
Severe(%)		26 (32.50)	30 (88.24)

Table S2. The information of 56 patients after the PCR test results turned negative.

	PF		Non-PF
N(%)	30(53.6)		26(46.4)
KL-6 level	579 [389.19,818.64]		340.50 [264.50, 402.50]
KL-6>505	18		2
KL-6>647	14		0
	Reversable	Irreversible	
N(%)	10(33.3)	20(66.7)	
KL-6 level	481.22 [367.02, 565.75]	735.00 [433.57, 1046.50]	
KL-6>505	5	13	
KL-6>647	1	13	

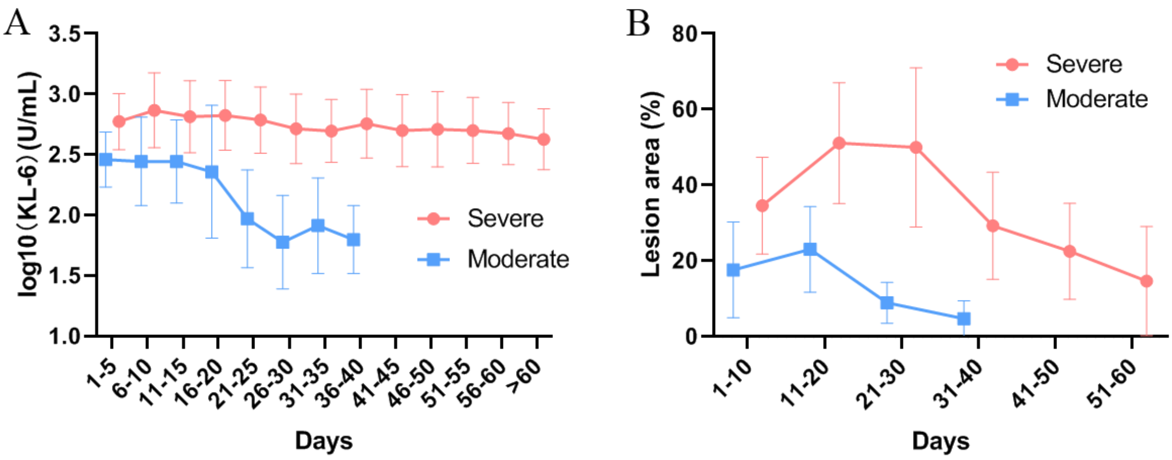

Figure S3. The trend of KL-6 and high-resolution CT (lesion area) in patients with severe and moderate COVID-19.

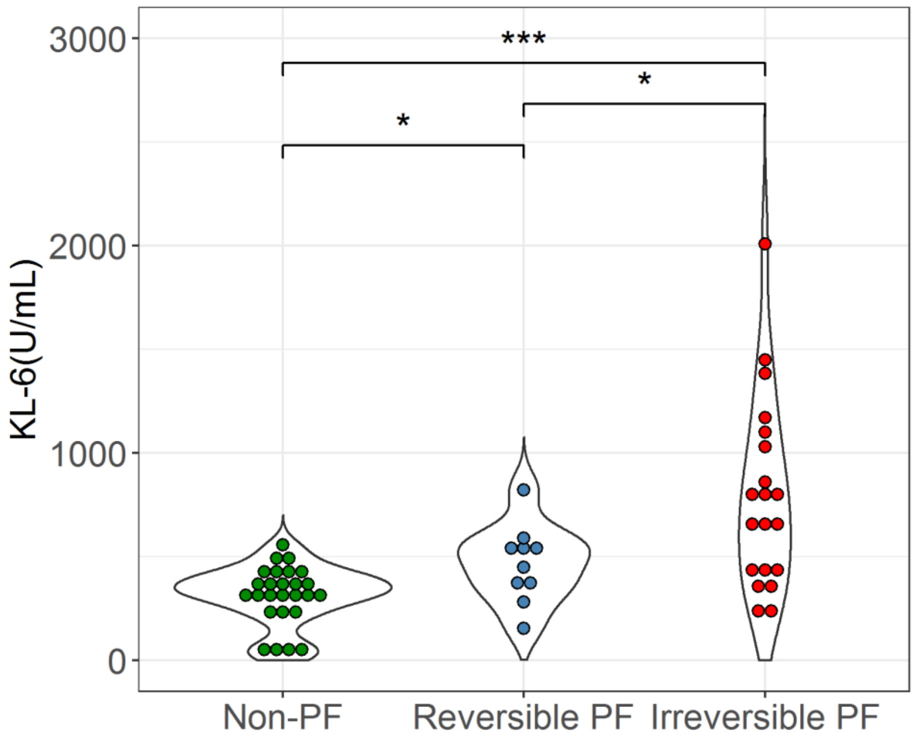

Figure S4. The difference of KL-6 after the PCR turn negative between COVID-19 patients without pulmonary fibrosis, with reversible pulmonary fibrosis and with irreversible pulmonary fibrosis.
